# Slow freezing cryopreservation of Korean bovine blastocysts with an additional sucrose pre-equilibration step

**DOI:** 10.3389/fvets.2024.1400899

**Published:** 2024-04-10

**Authors:** Seungki Jung, Hyeonseok Sul, Dongjin Oh, Yeon-Gil Jung, Joohyeong Lee, Sang-Hwan Hyun

**Affiliations:** ^1^Veterinary Medical Center and College of Veterinary Medicine, Laboratory of Veterinary Embryology and Biotechnology (VETEMBIO), Chungbuk National University, Cheongju, Republic of Korea; ^2^ET Biotech Co. Ltd., Jangsu, Republic of Korea; ^3^Institute of Stem Cell and Regenerative Medicine (ISCRM), Chungbuk National University, Cheongju, Republic of Korea; ^4^Department of Companion Animal Industry, Semyung University, Jecheon, Republic of Korea; ^5^Graduate School of Veterinary Biosecurity and Protection, Chungbuk National University, Cheongju, Republic of Korea

**Keywords:** sucrose, blastocyst, slow freezing, *in vitro* production, bovine

## Abstract

**Introduction:**

Embryo cryopreservation is a valuable technique used for preserving genetic resources for long periods. However, the survival rate of embryos is dependent on the method used. Therefore, in this study, we evaluated the efficiency of slow freezing method but with an additional dehydration step prior to freezing to overcome the formation of ice crystals.

**Methods:**

Oocytes collected from the ovaries of native Korean cattle subjected to *in vitro* fertilization were cultured for 7 days until the formation of expanded blastocysts. Before freezing, the blastocysts were placed in four pre-equilibration media: a control medium with no addition of sucrose, and three experimental media with the addition of 0.1, 0.25, and 0.5 M sucrose, respectively. Then, the pre-equilibrated embryos were frozen. Embryo survival and hatching rates were evaluated morphologically at 24, 48, and 72 h after thawing. Immunofluorescence staining, terminal deoxynucleotidyl transferase-mediated dUTP nick end labeling (TUNEL) assay, and gene expression analysis of the re-expanded blastocytes were examined 24 h after freeze–thawing.

**Results:**

The survival rate was significantly higher in the 0.1 M group than in the control group (*p* < 0.05), and the hatching rate at 72 h was significantly higher in the 0.25 and 0.5 M groups than in the control group (*p* < 0.05). TUNEL-positive cells were significantly lower in the 0.25 M group than in the control group (12.5 ± 0.9 vs. 8.3 ± 0.8; *p* < 0.05). The gene expression of *BCL2* associated X, heat shock protein 70 kDa, and aquaporin 3 in the 0.25 M group was significantly lower than that in the control group (*p* < 0.05).

**Conclusion:**

Our study revealed that treatment with 0.25 M sucrose before slow freezing improved the viability of bovine embryos after freeze–thawing.

## 1 Introduction

Currently, *in vitro* production (IVP) of embryos is a commercially successful approach for cattle breeding; the number of embryo transfers following IVP has increased since the establishment of the ovum pick-up-*in vitro* fertilization (IVF) system (1). In addition, the number of transplanted embryos produced *in vitro* is higher than that *in vivo* (1). The pregnancy rate of embryos produced after freeze-thawing is lower for embryos produced *in vitro* than for embryos produced *in vivo* (2, 3). Therefore, improvements in freezing methods are needed to increase the productivity of *in vitro* embryos after embryo transfer.

Cryopreservation methods, such as slow freezing, vitrification (4, 5), and low-temperature storage, are used to preserve cattle embryos (6). The slow freezing and vitrification methods can preserve genetic resources for long periods and have the advantage of embryos being transferred 7 days after the natural estrus cycle without synchronization. Vitrification has a faster cooling rate and does not form ice crystals, resulting in higher embryo survival and conception rates after embryo transfer (4, 5). However, vitrification involves the use of cryoprotectants at high concentrations, requiring their removal during the embryo thawing process (4, 7, 8). Therefore, this method is difficult to apply in field settings, because it requires skilled technicians, separate sterile facilities, and specific laboratory equipment at the embryo transfer site. In slow freezing, although relatively lower concentrations of cryoprotectants are used and in straw dilution and direct embryo transfer are practically applicable, the survival and conception rates of embryos in this method are relatively low (9), because of ice crystal formation within the embryos from the remaining water due to insufficient dehydration (10). Nevertheless, this process can be used at embryo transfer sites because the thawed embryos in the straws can be directly transferred into the recipients’ uterus (11). As intracellular ice formation has been identified as an important factor in slow frozen embryo survival, the possibility that improvements in embryo dehydration before slow freezing could improve the conception rate requires investigation. This aspect becomes particularly important as expanded bovine blastocysts produced *in vitro* with higher water content offer better conception rates (12).

Sucrose is a non-penetrating cryoprotectant used for removing intracellular water and protecting cells from osmotic shock in freezing media (7, 13). Bovine embryos were reportedly pre-equilibrated with sucrose before slow freezing; however, pretreatment did not improve embryo re-expansion and apoptosis rates (14). Bui-Xuan-Nguyen (15) pretreated bovine embryos with sucrose before slow freezing in a medium containing glycerol; however, viability did not improve. Usually, ethylene glycol (EG) is used as a freezing medium for direct embryo transfer owing to its low molecular weight and high permeability compared to glycerol. In addition, Voelkel and Hu (16) reported that embryos were slow freeze-thawed using EG, propylene giycol, dimethyl sulfoxide, and glycerol as cryoprotectants, and that embryos frozen in EG showed high viability.

The use of slow-freezing media supplemented with sucrose to freeze embryos is a well-known technique (17–19). However, there are limited reports on sucrose pre-equilibration using bovine embryos (14, 15). In this study, we aimed to evaluate embryo viability after slow freezing and thawing by inducing artificial shrinkage of the blastocoel cavity using different concentrations of sucrose medium before equilibration in a freezing medium containing EG. In addition, we investigated any differences in immunofluorescence staining, terminal deoxynucleotidyl transferase-mediated dUTP nick end labeling (TUNEL) assay results, and mRNA expression in blastocysts pretreated with sucrose at the optimal concentration compared with those in the control group.

## 2 Materials and methods

### 2.1 Oocyte collection and *in vitro* maturation

Ovaries from Korean native cattle (*Bos taurus coreanae*) were collected immediately after slaughter and transported to the laboratory in a physiological saline solution (0.9% NaCl) at 32°C. Cumulus–oocyte complexes (COCs) were collected via the aspiration of 2–8-mm follicles using an 18 G needle connected to a 10 mL syringe. The aspirated COCs with a homogeneous cytoplasm and at least three layers of cumulus cells were washed three times in OCM (IFP9611; Research Institute for the Functional Peptides, Yamagata, Japan). The selected COCs were then washed three times in HP-M199 (IFP971; Research Institute for the Functional Peptides) supplemented with 0.5 μg/mL follicle-stimulating hormone (Folltropin V; Bioniche Animal Health, Belleville, ON, Canada) and 5% fetal bovine serum (FBS; Access Biologicals, Vista, CA, United States) used as *in vitro* maturation (IVM) medium. The COCs were randomly cultured in 5-well dishes with 50 COCs per well (1 mL IVM medium per well) for 22 h at 38.9°C under 5% CO_2_ and 100% humidity.

### 2.2 *In vitro* fertilization and *in vitro* culture of embryos

*In vitro* fertilization was performed as reported by Kim et al. (6) with minor modifications. Frozen semen from Korean cattle were stored at −196°C in 0.5-mL straws until required. The straws were thawed in a 38°C water bath for 40 s. The thawed sperm were transferred into a 15-mL conical tube containing 1 mL of SPF45 and 1 mL of 80 SpermFilter® (Gynotec, Malden, Netherlands) and centrifuged at 600 *g* for 15 min. The precipitate was then transferred to a new centrifuge tube with 4 mL of IVF100 (IFP9630; Research Institute for the Functional Peptides) and centrifuged at 600 *g* for 10 min. 3.7 mL of supernatant was removed and 15 μL of sperm suspension (1 × 10^7^ cells/mL) was added to 50 μL of IVF100 drops, resulting in a final sperm concentration of 2 × 10^6^ cells/mL. Approximately 10–15 cumulus cell expanded oocytes were placed per drop and incubated at 38.9°C under 5% CO_2_ and 100% humidity for 6 h. The day of fertilization was labeled as day 0. At the end of IVF, the presumptive zygote was removed from the cumulus cells and sperm by pipetting. For *in vitro* culture (IVC), 10–15 presumptive zygotes were placed in a 50 mL drop of potassium simplex optimized medium and incubated for 7 days at 38.9°C under 5% CO_2,_ 5% O_2_, and 100% humidity.

### 2.3 Experimental design

#### 2.3.1 Experiment 1: survival and hatching rates of expanded bovine blastocysts exposed to various concentrations of sucrose prior to slow freeze–thawing

Expanded blastocysts developed 7 days after IVF were divided into four groups treated with 0 (control), 0.1, 0.25, and 0.5 M sucrose media. To investigate the effect of artificial shrinkage of the blastocoel cavity on embryos treated with sucrose before slow freezing, the survival rate was morphologically evaluated by the number of re-expanded blastocysts 24 h after thawing and the hatching rate at 24, 48, and 72 h.

#### 2.3.2 Experiment 2: influence of pre-exposure of bovine blastocysts to 0.25 M sucrose on different post-thaw viability measurements

Based on the total number of embryos hatched and embryo hatching/survival rates in Experiment 1, Experiment 2 was conducted using control and 0.25 M sucrose pretreatment groups. The total and apoptotic cell numbers of embryos thawed using each freezing method were confirmed using the TUNEL assay, and the total inner cell mass (ICM) number was determined using SOX2 antibody immunofluorescence. Finally, the effects of sucrose pretreatment before slow freezing on the gene expression levels of *BCL2* associated X (*BAX*), heat shock protein 70 kDa (*HSPA1A*), aquaporin 3 (*AQP3*), and placenta-specific 8 (*PLAC8*) in embryos after thawing were investigated.

### 2.4 Slow freezing

**Figure 1:**
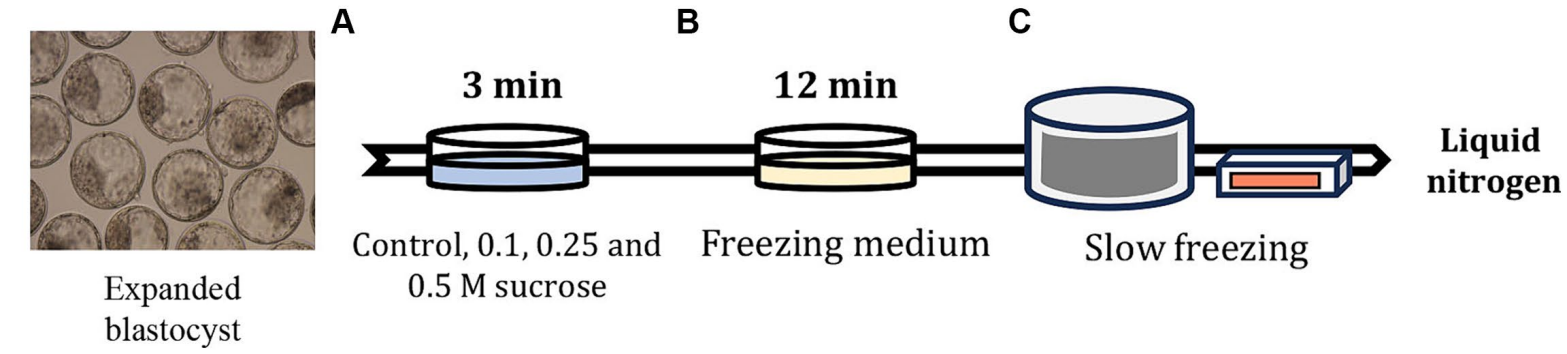
Slow freezing process after artificially shrinking the embryo blastocoel cavity using sucrose medium. **(A)** After 7 days of IVF, the expanded blastocysts were pre-equilibrated in Dulbecco’s phosphate buffered saline (DPBS) containing sucrose and 20% FBS for 3 min. **(B)** After confirming blastocoel cavity shrinkage, the embryos were equilibrated in DPBS containing 1.8 M EG for 12 min, and then the embryos were inserted in 0.25-mL straws and sealed. **(C)** The straws containing embryos were placed in a freezer maintained at −6°C and seeded after equilibration for 2 min. After 10 min, the straws were gradually frozen to −35°C at −0.3°C/min and stored in liquid nitrogen.

All experiments were performed using good-quality expanded blastocysts developed 7 days after IVF and were scored according to the International Embryo Technology Society guidelines (20). As shown in Figure 1, the expanded blastocysts were exposed to control, and 0.1, 0.25, and 0.5 M sucrose solution in Dulbecco’s phosphate buffered saline (DPBS; Gibco, Waltham, MA, United States) containing 20% FBS for 3 min at 20–25°C. The expanded blastocysts were washed three times in freezing medium without sucrose, containing 1.8 M EG (IFP9620; Research Institute for the Functional Peptides) to completely remove the sucrose pretreatment medium; the blastocysts were then equilibrated in the same freezing medium for 12 min at 20–25°C. During EG exposure, 5–10 expanded blastocysts per group were inserted in 0.25-mL straws (IMV Technologies, L’Aigle, France). Following exposure, the straws were placed in a freezer (FREEZE CONTROL®; Cryologic, Victoria, Australia) at −6°C. After 2 min, seeding was induced using a pair of tweezers to avoid supercooling. After another 10 min, the straws were frozen to −35°C at a rate of −0.3°C per min. After freezing, the straws were stored in liquid nitrogen for at least 1 week. The frozen straws containing the expanded blastocysts were thawed for 10 s in air and 30 s in 35°C water. The recovered expanded blastocysts were then washed three times in DPBS containing 10% FBS, diluted in IVC medium for 10 min, and cultured in the same medium for 72 h at 38.9°C under 5%CO_2_, 5%O_2_, and 100% humidity. Survival and hatching rates were determined based on the number of re-expanded and hatched blastocysts 24, 48, and 72 h after thawing.

### 2.5 Immunofluorescence analysis for surviving embryos after slow freeze–thawing

Immunofluorescence analysis was performed as reported by Lee et al. (21). Re-expanded or hatched blastocysts in 24-h culture after freeze–thawing were fixed with 4% (v/v) paraformaldehyde, washed with DPBS, and permeabilized with 0.5% (v/v) Triton X-100 for 30 min. The blastocysts were then co-incubated with a blocking solution and SOX2 primary antibody (sc-365823, 1:100; Santa Cruz Biotechnology, Santa Cruz, CA, United States) overnight at 4°C. After rinsing with washing medium (Tween20, Triton X-100, and DPBS), the expanded blastocysts were incubated with the secondary antibodies: goat anti-mouse IgG (H + L) Alexa FluorTM 488 (A11029, 1:200; Invitrogen Corporation, Carlsbad, CA, United States), donkey anti-rabbit IgG (H + L) Alexa FluorTM 594 (A21207, 1:400; Invitrogen) for 1 h at 20°C–25°C. The nuclei were stained with Hoechst-33342 and examined under an epifluorescence microscope using the ZEN 3.5 Blue Edition software (Zeiss, Germany).

### 2.6 TUNEL assay for surviving embryos after slow freeze–thawing

The TUNEL assay was performed as described by Lee et al. (21). To analyze apoptosis in the re-expanded and hatched blastocysts 24 h after freeze–thawing, the blastocysts were fixed with 4% paraformaldehyde for 1 h at 20–25°C and washed with DPBS. Permeabilization was performed using 0.1% Triton X-100 in 0.1% (w/v) sodium citrate for 1 h at 20–25°C. After washing with DPBS, the expanded blastocysts were stained with 45 mL of TUNEL-Label solution (Roche, Germany) containing 5 mL of TUNEL-Enzyme solution (Roche) for 1 h at 39°C in a dark, humidified atmosphere. Subsequently, the nuclei were stained with 5 μg/mL Hoechst-33342 for 10 min and the blastocysts were analyzed under an epifluorescence microscope.

### 2.7 Quantitative reverse transcription-PCR analysis for surviving embryos after slow freeze–thawing

mRNA was isolated from three replicates, each replicate containing five embryos. Quantitative reverse transcription-PCR (qRT-PCR) was performed as reported by Oh et al. (22). mRNA expression of four genes, *BAX*, *HSPA1A*, *AQP3*, and *PLAC8*, was analyzed using qRT-PCR with the primer sequences listed in Table 1. After recovery from freeze–thawing for 24 h, the re-expanded or hatched blastocysts were washed with DPBS and snap frozen sampled at −80°C prior to experimentation. The total RNA was extracted using TRIzol reagent (TaKaRa Bio, Inc., Otsu, Shiga, Japan) according to the manufacturer’s protocol and quantified by measuring the absorbance at 260/280 nm. The purity of the extracted RNA was 1.9; the RNA was stored at −80°C until use. Total RNA (1 μg) was converted to complementary DNA (cDNA) using the SuperScript IV VILO Master Mix (Thermo Fisher Scientific, Waltham, MA, United States). The synthesized cDNA, SYBR Premix Ex Taq (TaKaRa Bio, Inc.), and specific primers (Macrogen, Inc., Seoul, Republic of Korea) were used for qRT-PCR. mRNA expression was analyzed using the CFX96 Touch Real-Time PCR Detection System (Bio-Rad, Hercules, CA, United States). The cycling parameters were as follows: 40 cycles for 5 min at 95°C, 15 s at 95°C, 15 s at 56°C, and 30 s at 72°C. Relative quantification was performed by comparing threshold cycles (Ct) at a constant fluorescence intensity. Relative mRNA expression (*R*) was calculated using the equation *R* = 2^−[1Ctsample−1Ctcontrol]^ (23). The *R*-values were normalized to those of glyceraldehyde 3-phosphate dehydrogenase (*GAPDH*) in each blastocyst group (24).

**Table 1:** Primer sequences for quantitative reverse transcription PCR analysis.

Gene	Primer sequence	Product size (bp)	GenBank accession number
*BAX*	F: 5′-TGTCGCCCTTTTCTACTTTG-3′	204	NM_173894.1
	R: 5′-GCCACAAAGATGGTCACTGT-3′		
*HSPA1A*	F: 5′-GAGTCGTACGCCTTCAACAT-3′	194	NM_203322.3
	R: 5′-ATGATGGGGTTACACACCTG-3′		
*PLAC8A*	F: 5′-GACTGGCATCTTTGACTGCT-3′	195	NM_001025325.2
	R: 5′-GTCAGACACAGGCAATCCTT-3′		
*AQP3*	F: 5′-TTGGTCATCGGTACCTCAAT-3′	196	NM_001079794.1
	R: 5′-TCATGAGCTGGTACACGAAG-3′		
*GAPDH*	F: 5′-CTGGAGAAACCTGCCAAGTA-3′	186	NM_001034034.2
	R: 5′-GAGCTTGACAAAGTGGTCGT-3′		

### 2.8 Statistical analysis

Statistical analyses were performed using the Statistical Analysis System software (version 9.4; SAS Institute, Cary, NC, United States). Data were analyzed using the general linear model approach, followed by the mean separation method, with the least significant difference (*p* < 0.05) when there was a difference between treatments. Percentage data were arcsine-transformed prior to analysis to maintain the homogeneity of variance. Results are expressed as mean ± standard error of the mean.

## 3 Results

### 3.1 Effect of sucrose pretreatment on blastocyst survival after slow freezing and thawing

In Experiment 1, the embryos were freeze-thawed using sucrose of various concentrations to determine the optimal sucrose concentration for treatment before slow freezing. The blastocyst survival rate after slow freeze–thawing is shown in Table 2. The survival rate was higher in the 0.1 M group than in the control group (*p* < 0.05); however, no significant differences were observed between the 0.25 and 0.5 M groups. There were no significant differences in the hatching rates among the groups at 24 and 48 h. However, the hatching rate at 72 h was significantly higher (*p* < 0.05) in the 0.25 and 0.5 M groups than in the control group. The hatching rate at 72 h was not significantly different among the 0.1, 0.25, and 0.5 M groups. The proportion of hatched blastocysts among those that survived slow freezing and thawing was higher (*p* < 0.05) in the 0.25 M group than in the control group, and the proportion in the 0.1 and 0.5 M groups was not significantly different from that in the control and 0.25 M groups. Hence, we performed Experiment 2 using 0.25 M sucrose. The results suggested that pretreatment with 0.25 M sucrose could affect embryo recovery after thawing.

**Table 2:** Effect of sucrose on survival rate at 24 h and hatching rate of bovine blastocysts at 72 h after slow freezing and thawing.

Sucrose (M)	Survived (re-expanded) embryos/No. of Blastocysts (%)	No. of hatched blastocysts (%)			Embryo hatched/ Survived (%)
		24 h	48 h	72 h	
0 (Control)	59/65 (90.1 ± 4.5)^a^	5 (6.5 ± 2.6)	30 (43.6 ± 5.9)	38 (58.0 ± 6.4)^a^	38 (65.3 ± 7.2)^a^
0.1	64/65 (99.2 ± 0.8)^b^	13 (18.6 ± 8.1)	37 (53.8 ± 10.5)	48 (76.6 ± 6.1)^ab^	48 (77.1 ± 5.9)^ab^
0.25	62/65 (97.5 ± 2.5)^ab^	12 (17.1 ± 6.7)	34 (55.1 ± 8.2)	54 (87.6 ± 6.0)^b^	54 (89.6 ± 5.0)^b^
0.5	61/66 (95.4 ± 3.0)^ab^	16 (24.6 ± 9.7)	38 (59.4 ± 4.6)	50 (80.1 ± 5.6)^b^	50 (83.5 ± 3.9)^ab^

Seven replicates; data are presented as mean ± standard error. ^a,b^Values in the same column with different superscript letters are significantly different (*p* < 0.05).

### 3.2 Effects of sucrose pretreatment on ICM and trophectoderm of blastocysts after slow freezing and thawing

For Experiment 2 (0 and 0.25 M sucrose treatments), 24 h after thawing the embryos, we examined the ICM and TE using immunofluorescence analysis. No significant differences were observed in the total cell, ICM, or TE numbers between the groups. In addition, the ICM ratio of the total cell number showed no significant differences between the control and 0.25 M groups (*p* > 0.05, Table 3).

**Table 3:** Effect of sucrose on bovine blastocyst cells as determined using immunofluorescence analysis and TUNEL assay after slow freezing and thawing.

Sucrose (M)	Immunofluorescence analysis					TUNEL assay			
	No. of Blastocysts (No. of replicates)	Total cells	ICM	TE	ICM/TE cells (%)	No. of blastocysts (No. of replicates)	Total cells	Apoptotic cells	Apoptotic/total cells (%)
		Mean	Mean	Mean			Mean	Mean	
0 (Control)	13 (3)	171.0 ± 9.2	52.2 ± 7.1	118.8 ± 7.6	30.5 ± 3.3	24 (3)	171.1 ± 6.5	12.5 ± 0.9^a^	7.5 ± 0.6^a^
0.25	13 (3)	183.2 ± 7.1	55.3 ± 7.2	127.8 ± 5.2	30.2 ± 3.3	19 (3)	184.4 ± 8.7	8.3 ± 0.8^b^	4.6 ± 0.5^b^

Data are presented as mean ± standard error. ^a,b^Values in the same column with different superscript letters are significantly different (*p* < 0.05).

### 3.3 Effect of sucrose pretreatment on the apoptosis of blastocysts after slow freezing and thawing

To analyze apoptosis, the embryos were subjected to TUNEL assay 24 h after thawing. The total cell and apoptotic cell numbers in the blastocysts are shown in Table 3. No significant differences were observed in the total cell number between the control and 0.25 M groups. However, the number of apoptotic cells was significantly higher in the control group than in the 0.25 M group (*p* < 0.05).

### 3.4 Effect of sucrose pretreatment on gene expression levels in blastocysts after slow freezing and thawing

**Figure 2:**
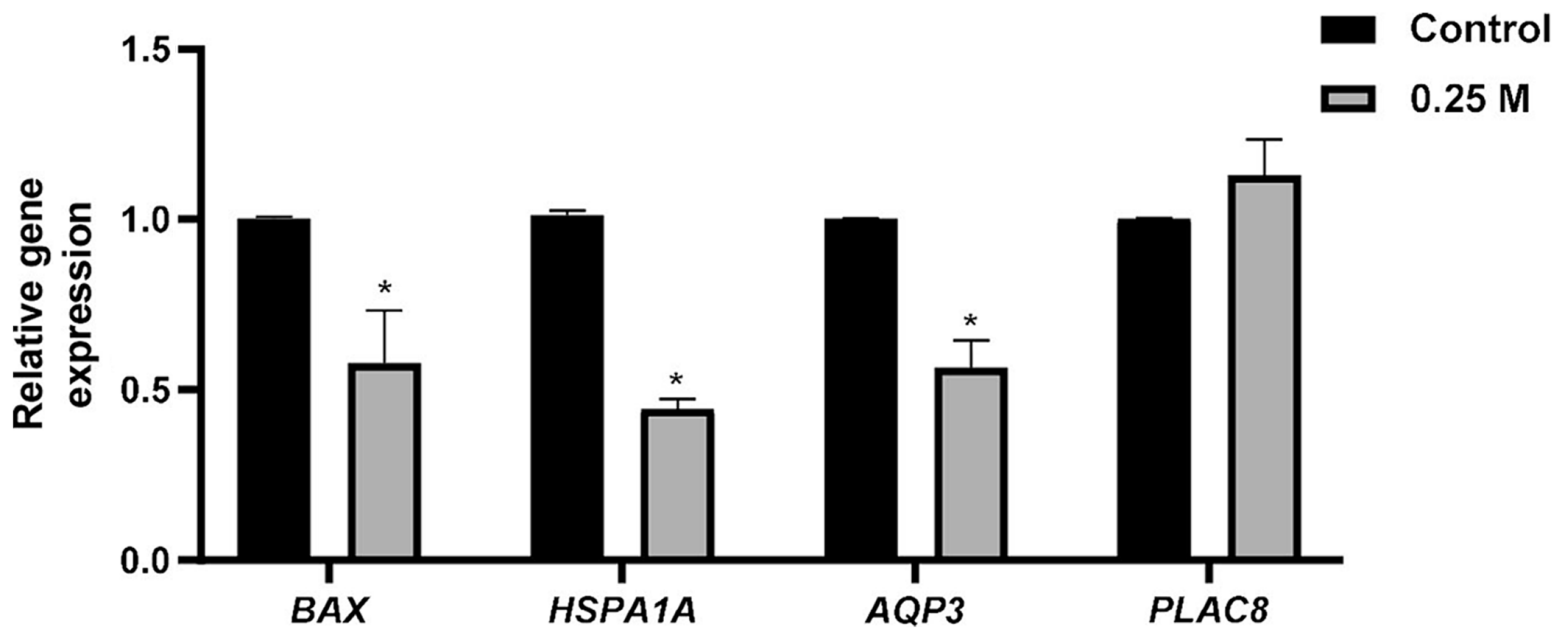
Effect of sucrose medium treatment before slow freezing of bovine blastocysts on the relative mRNA expression after thawing. *BAX*, BCL2 associated X; *HSPA1A*, Heat shock protein 70 kDa; *AQP3*, Aquaporin 3; *PLAC8*, Placenta specific 8. ^*^Different superscripts indicate significant differences for each gene (*p* < 0.05).

To analyze the effect of sucrose pretreatment on gene expression in embryos after freeze–thawing, qRT-PCR was performed. In the 0.25 M group, the relative mRNA expression of *BAX*, *HSP1A1*, and *AQP3* was significantly lower (*p* < 0.05) than that in the control group (Figure 2). However, the control and 0.25 M groups showed no significant differences in *PLAC8* expression (Figure 2).

## 4 Discussion

In this study, we investigated the effect of sucrose treatment on bovine embryos before freezing on the viability of the embryos after thawing. We demonstrated that embryos treated with sucrose before freezing had improved hatching rate after thawing. In addition, we demonstrated that sucrose treatment of bovine embryos prior to freezing decreased the transcript levels of apoptosis and cell stress related genes after thawing.

A slow freezing method for shrinking cattle embryos by adding sucrose to the freezing medium has been designed (9, 11, 17, 25). However, data on slow freezing methods using sucrose pretreatment prior to freezing are insufficient. Hence, in this study, we investigated blastocyst apoptosis and hatching rates upon pretreatment with various concentrations of sucrose before slow freezing. Iwayama et al. (26) reported no differences in the overall implantation rate when vitrification was performed after artificial shrinkage using a sucrose medium. However, high implantation rates were confirmed in embryos that recovered quickly after vitrification warming via sucrose medium pretreatment (26). In addition, Dang-Nguyen et al. (27) evaluated porcine oocyte quality using 0.2 M sucrose medium and Van Soom et al. (28) induced contraction of bovine morulae using 0.3 M sucrose medium; however, these treatments did not have a negative effect on viability. In this study, artificial shrinkage of the blastocoel cavity and blastomeres was induced using control, and 0.1, 0.25, and 0.5 M sucrose medium before slow freezing. We were unable to examine the effect of using sucrose medium on embryos without subsequent freezing; however, no adverse effects on embryos were identified after freeze–thawing. Additionally, we found that pretreating bovine embryos with 0.25 M sucrose resulted in higher hatching and hatching/survival rates at 72 h than in the control group after freeze–thawing. Thus, using the osmotic agent medium dehydrates the embryos and blastomeres. When the embryos are exposed to the freezing medium, a higher amount of the cryoprotectant penetrates them, possibly improving the viability of the embryos after slow freeze–thawing. Moreover, the blastocoel cavity could be artificially shrunk using a sucrose medium; it does not require special equipment or techniques and is easy to prepare sucrose medium.

The embryo does not fully recover within 24 h after thawing. We examined the hatching rate at 72 h after thawing to determine the possibility of implantation. However, in Experiment 2, the embryos were used 24 h after thawing to evaluate the quality of surviving embryos. Apoptosis maintains embryonic development and homeostasis, producing normal cells or removing abnormal cells; increased apoptosis is an important indicator for evaluating embryo quality. Apoptosis has been used to evaluate the quality of bovine (25), mouse (29), goat (30), equine (31), and human (29) embryos produced using the IVP system and that of *in vivo* and *in vitro* embryos. Additionally, vitrification and slow freezing methods damage embryonic cells by physical, chemical, and thermal factors during the freezing process; therefore, cell death is an important indicator of the effect of each freezing method on embryos (32, 33). In our study, only the control and 0.25 M groups were used to evaluate apoptosis, because the 0.25 M group showed the highest viability after freezing among the 0.1, 0.25, and 0.5 M groups. In addition, TUNEL staining showed no difference in the total number of cells between the groups evaluated 24 h after thawing, but the number of apoptotic cells was significantly lower in the 0.25 M group than in the control group. Compared to the control group, the 0.25 M group was confirmed to artificially shrink embryonic cells. The damage to blastomeres was assumed to be reduced by reducing ice crystal formation along with the dehydration of embryos. In future studies, the conception rate of frozen blastocysts after artificial shrinkage using 0.25 M sucrose before freezing should be examined.

The first lineage specification event during mammalian embryogenesis is the differentiation of the TE and ICM. The ICM develops into the hypodermis and ectoderm, and the TE develops outward from the fetal portion of the placenta. In human embryos, differences in cell numbers between the ICM and TE have been reported to reduce early embryo loss and improve implantation and birth rates (34). In cattle, the ICM and TE cell numbers are vary significantly depending on the culture system (35, 36). The ratio and distribution of these cells are a potential indicator of embryo quality and are considered crucial for implantation (37). However, in this study, no differences were observed in the number of ICM and TE cells between the control and 0.25 M groups. In the additional experiments, no differences were observed in TE cell numbers; however, confirming the embryo quality through the analysis of *INFT2*, which affects the conception rate, is necessary.

The relative mRNA expression in embryos after freeze–thawing has been widely reported. The relative mRNA expression levels in embryos vary according to culture conditions and time after freeze–thawing. *BAX* is a pro-apoptotic gene associated with apoptosis and a factor in determining embryo quality (38, 39). In this study, a significant difference was confirmed in *BAX* expression level between the control and 0.25 M groups. Together with TUNEL staining, this finding demonstrated that the number of positive cells in the 0.25 M group was smaller than that in the control group at the gene expression level. HSPs belong to a class of proteins called chaperones, whose expression is induced as a defense mechanism under all types of cellular stress; they are important cellular stress markers (40, 41). In addition, a previous study has observed an increase in *HSPA1A* level along with DNA fragmentation and apoptosis (42). Mori et al. (40) reported an increase in *HSPA1A* level in embryos under heat stress, indicating a decrease in embryo quality and viability. Park et al. (42) reported an increase in *HSP70* expression in embryos and a decrease in their viability based on the freezing method. In this study, *HSPA1A* expression was higher in the control group than in the 0.25 M group, suggesting that pretreatment with 0.25 M sucrose reduced expanded blastocyst stress. This result implies the possibility that pretreatment with sucrose before slow freezing can prevent damage to embryonic cells and reduce stress by dehydrating blastomeres and embryos. *AQP3* is a water molecule channel protein that allows water to flow across the membrane in the osmotic gradient direction, primarily expressed in epithelial tissues (43). *AQP3* expression levels in different culture conditions for bovine embryos (43, 44), mouse oocytes (45), and mouse embryos (46) have been investigated. Wang et al. (43) have reported that adding melatonin to the IVC medium significantly lowered the relative mRNA expression of *AQP3* compared with the control and increased survival after freeze–thawing. Similar results were obtained in this study; however, a previous study indicated that a decrease in the relative expression of *AQP3* adversely affected viability after freeze–thawing (47, 48). Hence, the difference in the culture system and increased *AQP3* expression are speculated indicators of rapid water influx after freeze–thawing and osmotic stress in embryos; further studies are needed in this field. Although *PLAC8* expression is not well established in cattle, its expression is related to placental development (49). High *PLAC8* expression in bovine embryos results in increased conception rates after embryo transfer (50); *PLAC8* expression is higher in the endometrium of pregnant cows than in that of non-pregnant cows (51). However, in this study, no difference was observed in *PLAC8* expression between the control and 0.25 M groups, because cell death and stress are reduced by *BAX* and *HSPA1A* expression, which has a beneficial effect on embryo survival and quality. However, embryo survival rates may not affect the conception rate, requiring additional embryo transfer experiments.

This study is limited in that it only demonstrated viability after freeze-thawing without embryo transfer using *in vitro* embryos. In addition, it did not quantify changes in embryos after treatment with sucrose medium. To overcome these limitations, further studies on conception rates in farmer’s fields by freeze-thawing bovine embryos pretreated with sucrose and direct embryo transfer are needed, and this study will help inform future frozen embryo transfer studies because embryo survival and conception rates are closely related (14, 52).

Our results indicate that artificially shrinking the blastocoel cavity using 0.25 M sucrose prior to slow freezing affects blastocyst viability and apoptosis after freezing–thawing. In addition, the relative mRNA expression of *BAX, HSPA1A*, and *AQP3* significantly decreased up on 0.25 M sucrose treatment. These results imply that the quality of bovine blastocysts can be improved using the slow freezing method aided by dehydration with a sucrose medium.

## Data availability statement

The datasets presented in this study can be found in online repositories. The names of the repository/repositories and accession number(s) can be found in the article/supplementary material.

## Ethics statement

Ethical approval was not required for the studies on animals in accordance with the local legislation and institutional requirements because only commercially available established cell lines were used.

## Author contributions

SJ: Conceptualization, Formal analysis, Investigation, Methodology, Validation, Writing – original draft, Writing – review & editing. HS: Formal analysis, Investigation, Methodology, Writing – original draft. DO: Formal analysis, Investigation, Methodology, Writing – original draft. Y-GJ: Formal analysis, Investigation, Methodology, Writing – original draft. JL: Conceptualization, Funding acquisition, Validation, Writing – original draft, Writing – review & editing. S-HH: Conceptualization, Funding acquisition, Validation, Writing – original draft, Writing – review & editing.
